# The Extent of Burn Injury Significantly Affects Serum Micro- and Macroelement Concentrations in Patients on the First Day of Hospitalisation

**DOI:** 10.3390/nu14204248

**Published:** 2022-10-12

**Authors:** Izabela Gutowska, Wojciech Żwierełło, Krzysztof Piorun, Marta Skórka-Majewicz, Dominika Maciejewska-Markiewicz, Patrycja Kupnicka, Irena Baranowska-Bosiacka, Bartosz Dalewski, Dariusz Chlubek

**Affiliations:** 1 Department of Medical Chemistry, Pomeranian Medical University, Powstańców Wlkp. 71 Street, 70-111 Szczecin, Poland; 2 West Pomeranian Center of Treating Severe Burns and Plastic Surgery, Niechorska 27 Street, 72-300 Gryfice, Poland; 3 Department of Human Nutrition and Metabolomic, Pomeranian Medical University, Broniewskiego 24 Street, 71-460 Szczecin, Poland; 4 Department of Biochemistry, Pomeranian Medical University, Powstańców Wlkp. 72 Street, 70-111 Szczecin, Poland; 5 Department of Dental Prosthetics, Pomeranian Medical University, Powstańców Wlkp. 72 Street, 70-111 Szczecin, Poland

**Keywords:** burn injurie, elements, hospitalization, serum

## Abstract

Burns exceeding 30% of total body surface area (TBSA) result in considerable hypovolemia coupled with the formation and release of inflammatory mediators, leading to subsequent systemic effects known as burn shock. Because of plasma exudation and the associated losses of large quantities of minerals, severe burns can lead to nutritional deficiencies and consequently disrupt homeostasis and metabolism of the entire body. The study group comprised 62 patients, who were divided into 3 groups according to the severity of burns. Serum samples were tested for concentrations of Ca, Mg, Mn, P, K, Zn, Cu, Fe, Se, Na, Cr, Ni, and Al. The mineral concentrations in serum of patients with burn injuries differ significantly from reference values, but this is not affected by the extent of the body burn. There are statistically significant decreases in serum concentrations of elements important for antioxidant protection (Zn, Cu, Se), and significant increases in the concentrations of toxic elements (Al and Ni), which may aggravate the effects associated with the state of burn shock. The Spearman rank correlation analysis did not reveal any statistically significant relationships between the serum concentrations of Mn, Ni, Al, K, Na, P, Mg, Zn, Se, Cr and the affected body surface area and severity of the burn—the values were at the lower end of the reference range. The obtained results indicate that proper nutrition, including elements replenishment, is extremely important in the recovery process of burn patients and time to nutrition is an important factor affecting patient survival after severe burn.

## 1. Introduction

A burn takes place when the skin comes into contact with a heat source [1]. It is associated with tissue injury caused by heat, friction, electricity, radiation, or chemicals [2]. Burn injuries vary widely, as does their severity. Morbidity and mortality rise with increasing burn surface area [3], while burn location, degree of temperature, and exposure time directly contribute to burn severity, showing a synergistic effect [4].

Any burn, even relatively minor, can have functional and aesthetic implications lasting throughout the patient’s lifetime. All efforts aimed at advancing our understanding of the problem of burns are gradually improving survival rates and quality of life for burn patients.

The skin is the largest organ of the human body, with a total area in an average adult of about 2 square meters. The structure of the skin and its components play an important role in wound healing, as a source of proliferating epithelial cells (keratinocytes) that migrate into the clot and wound bed. Loss of the physical barrier function of the skin enables invasion by harmful microorganisms, while increased fluid loss from the burned skin may impair the repair process of the wound, which begins even several hours after the injury [5,6].

In severely burned patients, one of the systemic reactions is an endocrine response with a dramatic increase in the secretion of hormones collectively labelled as stress hormones (catecholamine, glucagon, and cortisol) in the initial post-burn phase [7]. As a result, the body’s cardiovascular system is significantly affected, along with fluid and electrolyte balance.

The typical immediate response to thermal injury is plasma extravasation, followed by a sequence of hemodynamic events, including a drop in plasma volume, decreased cardiac and urine output on the one hand, and increased systemic vascular resistance (SVR) on the other. This results in reduced peripheral blood flow, producing elevated hemoglobin and hematocrit levels in burn insults [8,9]. Changes in the cardiovascular system also affect renal blood flow and glomerular filtration rate (GFR), which are reduced secondary to hypovolemia and reduced cardiac output [10].

Another characteristic body response to burns is edema formation [8,11], and the degree of edema development is determined by whether fluid resuscitation has been administered to the burned person. Following burn-induced plasma extravasation, additional extravasation occurs following resuscitation, since fluid support increases blood flow and capillary pressure, while the type and amount of administered fluid play a key role in determining the volume of edema [12,13].

Thermal injuries also have a major effect on cell membranes and on cellular transmembrane potential in both directly and indirectly traumatised cells. The decrease in membrane potentials triggers an increase in water and sodium content within the cells [14].

In addition, burns affect the gastrointestinal system, as evidenced by mucosal atrophy, reduced absorptive capacity, and increased intestinal permeability. The apoptosis of epithelial cells (enterocytes) occurs in proportion to the size of the burn, and as a result of the atrophy of the mucosa, the absorption function of the digestive system is impaired. This leads to defects in the absorptive function of the digestive system [15] and impaired uptake of not only fats (through the changes in the activity of pancreatic lipase) [15], proteins and carbohydrates but also vitamins and minerals, whose normal metabolism is beneficial following burn injury. They are important for normal immune processes and wound healing in severe burns because endogenous antioxidant activity is highly dependent on adequate micronutrient concentrations [16].

Acute deficiencies of micro- and macroelements are evident in burns exceeding 20% TBSA (total body surface area), which can be attributed to large exudative burn wound losses containing significant quantities of Fe, Cu, Se, and Zn. Early intravenous supplementation has become a recognised strategy recommended by the American and European burn organisations (European Society for Clinical Nutrition and Metabolism, European Burns Association, American Burn Association), as it decreases infectious complications and improves wound healing. Frequent determination of serum concentrations of these minerals can help detect pathologically low levels [17].

Maintaining normal body metabolism requires qualitative and quantitative contributions of many micro- and macroelements. Because of plasma exudation and the associated losses of large quantities of minerals, severe burns can lead to nutritional deficiencies and consequently disrupt homeostasis and metabolism of the entire body. Moreover, initiation of inflammatory and free radical processes leads to the progression of oxidative stress, the suppression of which largely depends on an adequate supply of mineral elements. As an additional risk, mineral deficiencies in burns may accelerate bone metabolism and lead to the release of mineral reservoirs from hard tissues, containing both essential and toxic elements which can pass into the serum. Their concentrations may increase temporarily and exert synergistic adverse effects on the patient’s body. Hence, the aim of the present study was to investigate the content of macro- and microelements in the blood serum of patients hospitalised for burn injury on the first day of hospitalisation.

## 2. Materials and Methods

### 2.1. Patients

The study group comprised 62 patients (13 women “W” and 49 men “M”) of the West Pomeranian Centre for Severe Burns and Plastic Surgery, Medicam Hospital in Gryfice, Poland. Based on the severity of burns, the patients were divided into 3 groups, as shown in Table 1 (classification according to [18]). The mean age of the patients was 48.1 years, SD = 16.8 years (group 1: mean = 40.8, SD = 14.3; group 2: mean = 46.3, SD = 16.6; group 3: mean = 51, SD = 17.1).

### 2.2. Collection of Material from Patients

Blood samples for biochemical tests were taken from patients on an empty stomach, upon admission to the West Pomeranian Centre for Severe Burns and Plastic Surgery. Blood for testing was drawn from a fresh venipuncture. All parameters were determined at the Department of Laboratory Diagnostics at the Gryfice hospital. The laboratory is equipped with modern, automatic analyzers and devices, connected with a two-way Laboratory Information System. This system supervises analytical processes—from automatic registration of orders and samples (barcodes), through calibration, control and determinations with automatic and manual validation, to the final result printout and full data and sample archiving. The laboratory participates in quality checks confirmed by current certificates (since 2002 in two international quality checks: RIQAS—Great Britain and QCS—Germany).

The following parameters were measured in the whole study group: total protein, albumin, aspartate aminotransferase (AST), alanine aminotransferase (ALT), vitamin D, creatinine, glucose, C-reactive protein (CRP), procalcitonin, IL-6, as well as complete blood count. After completing all biochemical tests, 2 mL of serum from each patient was recovered and immediately preserved at −80 °C until elemental analysis.

### 2.3. Determination of Mineral Content in Blood Serum by ICP-OES Method

Serum samples from patients were analysed for the concentrations of: Ca, Mg, Mn, P, K, Zn, Cu, Fe, Se, Na, Cr, Ni and Al using inductively coupled plasma optical emission spectrometry (ICP-OES, ICAP 7400 Duo, Thermo Fisher Scientific, Waltham, MA, USA) equipped with a concentric nebuliser and a cyclonic spray chamber.

Serum samples (1 mL) were subjected to a microwave decomposition procedure using a microwave digestion system (MARS 5, CEM). To this end, samples were transferred into clean polypropylene tubes, adding 1 mL of 65% HNO_3_ (Suprapur, Merck, Kenilworth, NJ, USA) and 1 mL of unstabilised 30% H_2_O_2_ (Suprapur, Merck, Kenilworth, NJ, USA). The samples were then transferred into Teflon vessels and placed in the microwave digester. The digestion process was carried out in two steps: step one, lasting 15 min during which the samples were gradually heated to 180 °C, and step two, lasting 20 min during which the temperature was maintained at 180 °C. The resulting digests were diluted 20-fold by adding an appropriate amount of deionised water (Direct Q UV, Millipore, approx.18.0 MΩ).

An internal standard—Yttrium (final concentration in the sample 0.5 mg/L) and 1 mL of 1% Triton (Triton X-100, Sigma, St Louis, MO, USA) was added to 0.5 mL of each sample. The samples were diluted with 0.075% HNO_3_ (Suprapur, Merck) up to the final volume of 10 mL, and stored in the fridge (4–8 °C) until analysis. The blank test was prepared according to the same procedure, with the study sample replaced by 0.25 mL of nitric acid (V).

The calibration curve was prepared using multi-element standard solutions (ICP multi-element standard solution IV, IX and XVI, Merck). All solutions were made using deionised water (Direct Q UV, Millipore, approx.18.0 MΩ). The following wavelengths (nm) were used in the analysis: (Ca) 315.879; (Mg) 279.553; (Mn) 257.610; (P) 178.766; (K) 279.553; (Zn) 202.548; (Cu) 224.700; (Fe) 238.204; (Se) 196.090; (Na) 589.592; (Cr) 267.716; (Ni) 232.003; (Al) 167.079.

The results obtained for the study group were compared with the reference values for mineral content in serum.

### 2.4. Statistical Analysis

The results were analysed using the Statistica 10.0 software package (StatSoft, Kraków, Poland). The arithmetic mean ±SD was calculated for each of the studied parameters. The distribution of results for each variable was tested using the Shapiro-Wilk W test for normality. Since most distributions deviated from a normal distribution, non-parametric tests were used in further analyses. Differences between the studied groups were evaluated by the non-parametric Mann-Whitney U test, with *p* ≤ 0.05 adopted to indicate statistical significance. The Spearman rank correlation test was used to measure the degree of correlation. P-values below 0.05 were considered statistically significant. Non-significant results are reported as NS (not significant) rather than a *p*-value.

## 3. Results

Table 2 summarises the mean determinations of biochemical parameters in patient groups divided according to burn severity. Tests were performed at a commercial diagnostic laboratory on the first day of hospitalisation.

In the analysis of the results of biochemical tests, it was observed that burn severity was inversely associated with blood levels of total protein and albumin. The values observed in patients with minor and moderate burns were at the lower end of the reference range, while in patients with severe burns they were just below normal. The differences between patients with minor and severe burns for total protein (*p* = 0.044) and between patients with minor vs. severe and moderate vs. severe burns for albumin (*p* = 0.0007 and *p* = 0.0044, respectively) were statistically significant.

The results of statistical analysis showed that blood levels of AST and ALT increased in direct proportion to burn severity. The values observed in patients with minor and moderate burns were at the upper end of the reference range, while in patients with severe burns they were clearly above the upper limit of the normal range for AST. The differences in groups 1 (minor burns) vs. 3 (moderate burns) and 2 (moderate burns) vs. 3 (severe burns) were statistically significant for AST (*p* = 0.018 and *p* = 0.039 respectively) and ALT (*p* = 0.013 and *p* = 0.039 respectively).

With respect to creatinine and glucose, an increase in burn severity was also associated with an increase in blood levels of these parameters. The observed creatinine values in all study groups were within the reference range, while glucose levels were above the upper limit of the reference range. For all patient groups, the differences were not statistically significant.

CRP and IL-6 concentrations in all study groups were at similar, markedly elevated levels (more than 10x for CRP) relative to the upper limit of the reference range. Differences between the study groups for CRP were not statistically significant, whereas for IL-6 they were statistically significant between group 1 (minor burns) vs. 2 (moderate burns) (*p* = 0.024) and group 1 (minor burns) vs. 3 (moderate burns) (*p* = 0.039).

Hematocrit and hemoglobin levels in all study groups were within the reference range. A trend was observable where the values of both parameters increased together with burn severity. The differences between patients with minor and patients with severe burns were statistically significant (*p* = 0.004 for hematocrit and *p* = 0.007 for hemoglobin).

The serum samples recovered after biochemical tests were used to determine the concentrations of Ca, Mg, Mn, P, K, Zn, Cu, Fe, Se, Na, Cr, Ni, and Al by way of inductively coupled plasma optical emission spectrometry (ICP-OES, ICAP 7400 Duo, Thermo Scientific). The arithmetic mean ±SD was calculated for each of the studied parameters. The results are presented as bar graphs, including the reference values (ref.) for each element. The assessment was made for differences between the study groups: group 1—minor burns, group 2—moderate burns, group 3—severe burns (according to [18]).

**Figure 1:**
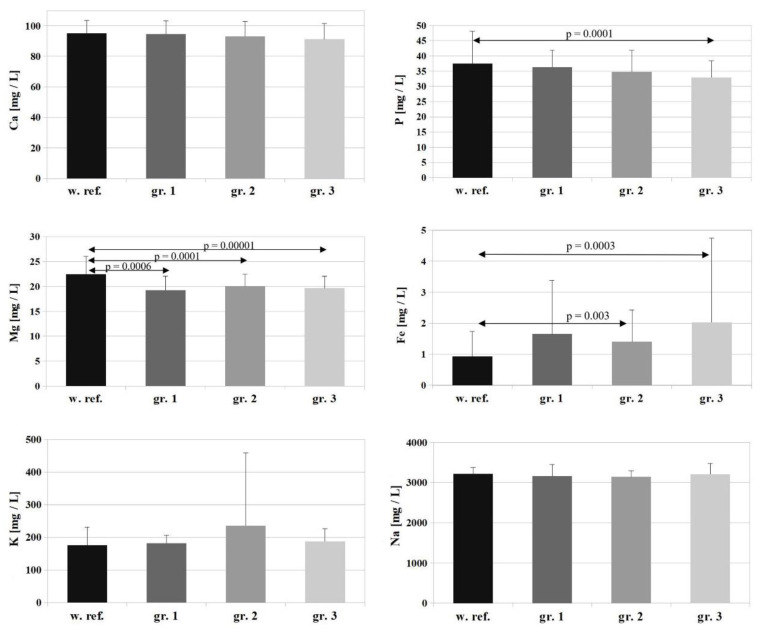
Comparison of reference values for Ca, P, Mg, Fe, K and Na and their concentrations in the serum of patients on the first day of hospitalization. w.ref.–reference value; gr–group.

The reference value for Ca is 95 ± 8.5 [mg/L], and the Ca levels determined in the study groups amounted to 94.6 ± 8.7 [mg/L], 93.2 ± 9.6 [mg/L], and 91.2 ± 10.4 [mg/L] (groups 1, 2, 3, respectively)—Figure 1. There were no changes in patients’ serum Ca levels corresponding with burn surface area (*p* = 0.188) or severity (*p* = 0.838). The values were at the lower end of the reference range, and the differences between the study groups were not statistically significant.

The reference value for Mg is 22.5 ± 3.5 [mg/L], and the Mg concentrations determined in the study groups were: group 1 (patients with minor burns)—19.29 ± 2.8 [mg/L], group 2 (patients with moderate burns)—20.09 ± 2.4 [mg/L], group 3 (patients with severe burns)—19.66 ± 2.4 [mg/L]. A statistically significant decrease in serum Mg was observed relative to the reference value in all study groups (*p* = 0.0006 for group 1, *p* = 0.0001 for group 2 and *p* = 0.00001 for group 3). The differences between the individual groups were not statistically significant (Figure 1).

For P, the reference value is 37.5 ± 10.6 [mg/L], and the following P concentrations were determined in the study groups: group 1—36.3 ± 5.6 [mg/L], group 2—34.8 ± 7.1 [mg/L], group 3—32.9 ± 5.5 [mg/L]. Serum P levels followed a slight downward trend corresponding with increasing surface area and severity of the burn, but the differences between individual study groups were not statistically significant (Figure 1).

For K (Figure 1), the reference value is 176 ± 55.2 [mg/L], and K concentrations determined in the study groups amounted to: group 1—182 ± 23.8 [mg/L], group 2—234.8 ± 223.3 [mg/L], group 3—187.3 ± 39.1 [mg/L]. There were no statistically significant changes in serum K levels relative to the reference value in any of the study groups or between study groups.

In the case of Na, the reference value is 3218.5 ± 161.9 [mg/L], and Na concentrations determined in the study groups amounted to: group 1—3161.9 ± 290.9 [mg/L], group 2—3142.3 ± 149.8 [mg/L], group 3—3209.9 ± 268.9 [mg/L]. There were no statistically significant changes in serum Na levels relative to the reference value in any of the study groups or between study groups (Figure 1).

The reference value for Fe is 0.925 ± 0.81 [mg/L], and Fe concentrations determined in the study groups were: group 1—1.653 ± 1.72 [mg/L], group 2—1.403 ± 1.02 [mg/L], group 3—2.031 ± 2.71 [mg/L]. A marked increase in serum Fe levels was observed in the patients relative to the reference value in all of the studied groups, but statistical significance was shown only for groups 2 and 3 (*p* = 0.03 for group 2 and *p* = 0.003 for group 3) (Figure 1).

**Figure 2:**
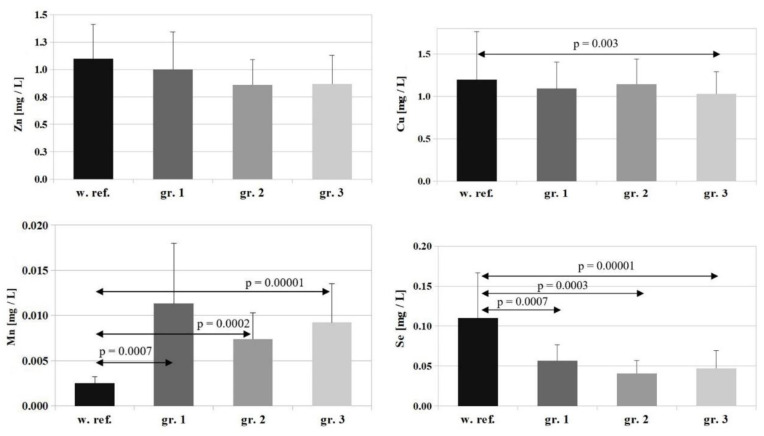
Comparison of reference values for Zn, Cu, Mn and Se and their concentrations in the serum of patients on the first day of hospitalization. w.ref.–reference value; gr–group.

In the case of Zn, the reference value is 1.1 ± 0.31 [mg/L], and the concentrations of this element determined in the study groups amounted to: group 1—1.0 ± 0.34 [mg/L], group 2—0.86 ± 0.29 [mg/L], group 3—0.87 ± 0.26 [mg/L]. A marked, but statistically insignificant, decrease in serum Zn levels was observed in all the study groups compared to the reference value (with the lowest serum Zn levels in patients in groups 2 and 3). The differences found between the individual groups were not statistically significant (Figure 2).

The reference value for Cu is 1.2 ± 0.56 [mg/L], and Cu concentrations determined in the study groups were: group 1—1.09 ± 0.31 [mg/L], group 2—1.14 ± 0.29 [mg/L], group 3—1.03 ± 0.26 [mg/L]. A slight decrease in serum Cu levels was observed in all study groups relative to the reference value, but the differences were statistically significant only for group 3 (*p* = 0.003). The differences between the individual groups were not statistically significant (Figure 2).

The reference value for Se is 0.11 ± 0.05 [mg/L], and Se concentrations determined in the study groups amounted to: group 1—0.056 ± 0.019 [mg/L], group 2—0.040 ± 0.016 [mg/L], group 3—0.046 ± 0.022 [mg/L]. A marked and statistically significant decrease in serum Se levels was observed in all the study groups compared to the reference value (*p* = 0.0007 for group 1, *p* = 0.0003 for group 2, and *p* = 0.00001 for group 3). The differences between the individual groups were not statistically significant (Figure 2).

The reference value for Mn is 0.0025 ± 0.0007 [mg/L], and Mn concentrations determined in the study groups were: group 1—0.0113 ± 0.0066 [mg/L], group 2—0.0074 ± 0.0029 [mg/L], group 3—0.0092 ± 0.0043 [mg/L]. A marked and statistically significant increase in serum Mn levels was observed in all study groups compared to the reference value (for group 1—*p* = 0.0007, for group 2—*p* = 0.0002, for group 3—*p* = 0.00001) (Figure 2).

**Figure 3:**
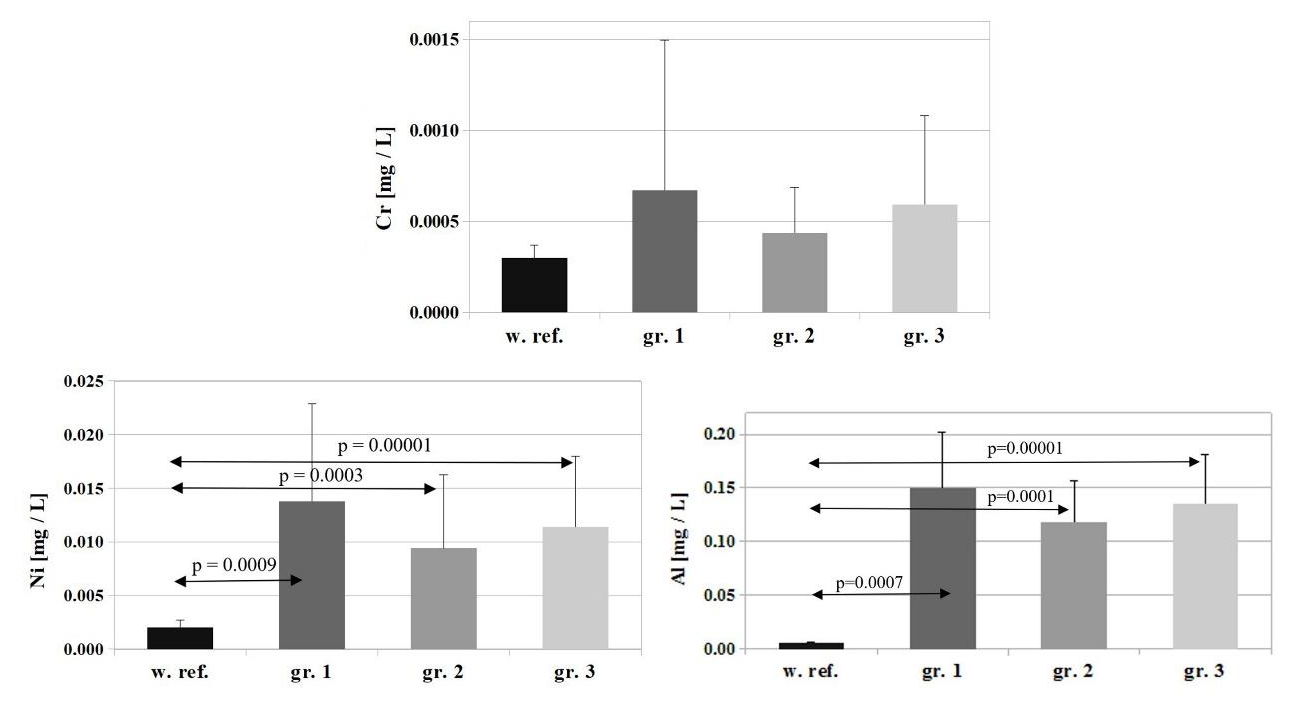
Comparison of reference values for Cr and permissible serum limit for Ni and Al and their concentrations in the serum of patients on the first day of hospitalization. w.ref.–reference value; gr–group.

For Cr, the reference value is 0.0003 ± 0.00007 [mg/L], and Cr concentrations determined in the study groups amounted to: group 1—0.0007 ± 0.0008 [mg/L], group 2—0.0004 ± 0.0002 [mg/L], group 3—0.0006 ± 0.0004 [mg/L]. A marked, but statistically insignificant, decrease in serum Cr levels were observed in all the study groups compared to the reference value (Figure 3).

The permissible serum limit for Ni is 0.002 ± 0.0007 [mg/L], and its concentrations determined in the individual study groups amounted to: group 1—0.014 ± 0.009 [mg/L]; group 2—0.009 ± 0.006 [mg/L]; group 3—0.011 ± 0.006 [mg/L]. A marked and statistically significant increase in serum Ni levels was observed in all the study groups relative to the reference value (*p* = 0.0009 for group 1, *p* = 0.0003 for group 2, and *p* = 0.00001 for group 3). The differences between the individual groups were not statistically significant (Figure 3).

Lastly, the permissible value for Al in serum is 0.006 ± 0.0007 [mg/L], and its concentrations determined in the study groups were: group 1—0.15 ± 0.051 [mg/L], group 2—0.118 ± 0.037 [mg/L], group 3—0.135 ± 0.046 [mg/L]. A marked and statistically significant increase in serum Al levels was observed in all the study groups relative to the permissible limit (*p* = 0.0007 for group 1, *p* = 0.0001 for group 2, and *p* = 0.00001 for group 3). The differences between the individual groups were not statistically significant (Figure 3).

Table 3 presents the results of the Spearman rank test for biochemical parameters and mineral concentrations in serum. The following correlation strength ranges were used in the interpretation: 0–0.2—very weak, 0.2–0.4—weak, 0.4–0.6—moderate, 0.6–0.8—strong, 0.8–0.9—very strong. All significant correlations represented weak relationships and the exact figures can be found in the discussion of results for individual minerals.

There was a negative correlation between serum Ca and hemoglobin levels (R = −0.241, *p* = 0.042), and a positive correlation with albumin concentration (R = 0.271, *p* = 0.025). 

For P, significant negative correlations were noted between serum levels of this element and IL-6 (R = −0.334, *p* = 0.009) and creatinine (R = −0.278, *p* = 0.019), as well as significant positive correlations with albumin (R = 0.289, *p* = 0.017) and total protein (R = 0.258, *p* = 0.043). The relationship with hemoglobin indicated a slight negative trend (R = −0.204, *p* = 0.088). In turn, a significant positive correlation was demonstrated between serum Mg and albumin levels (R = 0.205, *p* = 0.036), and a positive trend for ALT (R = 0.205, *p* = 0.088). For Zn, there was a statistically significant negative correlation between its serum level and glucose (R = 0.332, *p* = 0.012), and for Cr a negative trend was noted with respect to serum ALT (R = −0.225, *p* = 0.066).

The Spearman rank correlation analysis did not reveal any statistically significant relationships between the serum concentrations of Mn, Ni, Al, K, Na, P, Mg, Zn, Se, Cr and the affected body surface area and severity of the burn—values were at the lower end of the reference range.

## 4. Discussion

Maintaining normal metabolism of numerous vitamins and trace elements is beneficial after burn, as they are important for normal immune processes and wound healing. Major burns trigger severe oxidative stress, which combined with the substantial inflammatory response contributes to the depletion of endogenous antioxidants, which are highly dependent on adequate micronutrient concentrations [16].

Acute micro- and macroelement deficiencies are evident in burns exceeding 20% TBSA. They can be attributed to large exudative burn wound losses containing significant quantities of Fe, Cu, Se, and Zn. Delivering early intravenous supplementation has become an established strategy, resulting in fewer infectious complications and improved wound healing; it is recommended in both North America and Europe. Weekly determination of mineral levels should be performed at the very least in patients with burns greater than 40% TBSA. In severe burns, such testing has been shown to detect pathologically low values [17].

Among the essential minerals is zinc, playing a crucial role in wound healing, lymphocyte function, DNA replication and protein synthesis [19]. Iron acts as a cofactor for oxygen-carrying proteins, while Se enhances cell-mediated immunity [16]. Copper is critical for the formation of mature, organised collagen, and copper deficiency has been linked to cardiac arrhythmias, impaired immunity, and worse outcomes after burns [20].

Reduced levels of vitamins A, C, and D, as well as Fe, Cu, Se, and Zn have been shown to adversely affect wound healing rates and skeletal muscle metabolism, also leading to impaired immune function [21,22]. Vitamin A accelerates wound healing by stimulating epithelial growth, and vitamin C promote collagen maturation and cross-linking [23]. Vitamin D contributes to bone density and its deficiency after burns has been observed, but the exact role and optimal dose after severe burns remain unclear.

Pediatric burn patients may suffer significant dysfunction in Ca and vitamin D homeostasis for a number of reasons. Children with severe burns have increased bone resorption, osteoblast apoptosis, and urinary excretion of Ca. In addition, burned skin is unable to synthesise normal amounts of vitamin D3, leading to further disruption of Ca and vitamin D levels. A study of children with burns showed that supplementation with a multivitamin containing 400 IU of vitamin D2 failed to correct vitamin D deficiency [24].

### 4.1. Calcium

Measurement of total Ca levels is less useful clinically than testing its physiologically active fractions, free and ionised calcium. In a study of burn patients, Fenton et al. [25] observed hypocalcemia, though it was generally limited to low total Ca. On average, free or ionised calcium were in the normal range, and total and free Ca were weakly correlated with burn severity, suggesting the loss of several forms of Ca in severe burn injury. An algorithm was developed to calculate free Ca from total Ca and albumin. However, inter-patient variables affect the outcome of the calculation to such an extent that the algorithm should not replace the actual test [25].

Burns are often accompanied by the so-called “bone loss” phenomenon. It is associated with elevated blood cytokine levels (mainly IL-1β and IL-6) in connection with the inflammatory response, which stimulates the parathyroid glands to upregulate membrane-bound Ca-sensing receptor (CaR) [26,27]. This leads to reduced circulating Ca and reduced parathyroid hormone (PTH) secretion, resulting in renal calcium wasting, a mechanism that has been demonstrated in children [28]. Additionally, the inflammatory response in synergy with the stress response acts directly on bone, causing an increase in proinflammatory cytokines and endogenous glucocorticoids. Stimulated production of osteoblast RANK ligand increases bone resorption. However, when the stress response persists, osteoblasts and probably also osteocytes undergo apoptosis and bone turnover is markedly reduced, inhibiting Ca uptake from the blood. The situation may be additionally aggravated by the relatively common Zn deficiency and the associated conformational changes of calmodulin and/or calcium channels, resulting in reduced gastrointestinal Ca absorption [28].

In a study of the metabolic response to burn injury, it was found that children and adults had different calcemic response. In adult patients, circulating ionised Ca levels in the blood tend to be normal or even mildly elevated, and the same is true for PTH [29]. The reason for this discrepancy is unclear, but one possibility could be that cytokine-mediated regulation of CaR is age-dependent. The small number of studies on ionised Ca and PTH concentrations in children and adults with burn injury provide limited but largely consistent data suggesting a different set of responses involving Ca, CaR, and PTH to inflammatory and stress-related stimuli [30].

The absence of significant deviations in Ca levels in the studied patient groups relative to reference values may support the theory that serum Ca levels do not decline in adult patients and that cytokine-mediated CaR regulation is age-dependent.

### 4.2. Magnesium

The results presented in this paper point to a slight decrease in serum Mg levels relative to the reference value across all the studied groups. However, all results were at the lower end of normal, and the differences between groups were not statistically significant.

Mg deficiency in burn patients has been reported in several studies. Researchers [31] estimated serum magnesium on day 10 after a burn injury and found it to be low. Hypomagnesemia was observed in nearly 50% of cases, while more than 30% had magnesium deficiency syndrome. The same research team found no significant correlations between patient groups, the severity of burn injuries and Mg levels. Hypomagnesemia was treated as soon as detected, and it took 4–5 days of treatment for Mg levels to return to normal [31]. In the other study, severe Mg deficiency was noted in 8 out of 20 patients with thermal injuries. Decreased level of magnesium in serum was observed in both very early and late stages of burn treatment and was presenting with physical symptoms like muscle cramps and tremor and psychiatric symptoms including hallucinations, and depression [32]. Berger et al. reported a strong relationship between the development of hypomagnesemia and the treatment of burn patients with aminoglycoside, the latter being a common complication of this type of antibiotic therapy observed in a number of studies [33]. Likewise, another researchers found that magnesium deficiency persists into the recovery period of treatment [34]. Dissimilarly, Lafargue et al. reported that hypomagnesemia was temporary, usually returning to values considered normal from day 3 [35].

Most of the body’s total Mg content is stored in bone, muscle and soft tissues, while blood serum contains only about 1%, meaning that serum determinations unfortunately fail to accurately reflect the actual body Mg status. This element is involved in many biological processes, including nerve conduction, active potassium and calcium transport, thus regulating blood pressure and pulse. It plays a role in the regulation of muscular contraction, glucose metabolism and processes like energy production and protein synthesis [36]. Serum Mg homeostasis depends on the interplay of intestinal absorption and renal excretion, and hence a low Mg level may be attributable to excessive wasting (through exudation or excretion in urine), malabsorption or inadequate nutrition. Increased magnesium losses through the kidneys may be caused by glomerular necrosis as a result of hypovolemia and shock during the acute phase of burn injury. Renal Mg excretion can also be stimulated by endocrine abnormalities, like high cortisol and aldosterone levels, which are often observed in burn injuries [34]. Further, it has been suggested that the hypermetabolic response of burns may be implicated in Mg deficiency, due to the increased requirement for magnesium and its uptake into the cells. The elevated rates of metabolism observed in burn patients entail increased energy requirements of cells and may therefore cause higher intracellular magnesium uptake. It has also been hypothesised that a deficiency of magnesium, may block intracellular cyclic AMP syntesis in parathyroid cells, inhibiting the secretion of parathyroid hormone [37].

The results of the present study are consistent with previously reported data. Mg deficiency is multifactorial in the acute phase of burn injury and may persist during the recovery period. Therefore, it is important to emphasise the importance of controlling serum Mg levels from the first hours of patient admission following burn injury and regular monitoring throughout the recovery period.

### 4.3. Phosphorus

In this study, serum P levels were observed to follow a slight downward trend which got stronger with increasing surface area and severity of the burn, but the differences between individual study groups were not statistically significant. The decrease observed on the first day after burn injury may predict the onset of hypophosphatemia at a later time. The latter usually occurs around day three post-burn, reaching the lowest level around day five/six [38].

A burn injury causes a relatively rapid decrease in serum P concentration. Significant losses of this element have been shown via both urine and burn wound exudates [38]. Despite aggressive supplementation with P-containing preparations, normalisation of its serum levels is rarely observed before day 10 after burn injury. Exogenous administration of epinephrine additionally accelerates the onset of hypophosphatemia. This may suggest that the release of catecholamines is another contributor to the rapid decrease in serum P concentrations, and that in the early post-burn period the metabolic response with an increase in catecholamines, glucagon, and cortisol is a standard body response to trauma [39].

Phosphorus in the form of phosphate esters is a component of purine nucleotides, which play an important role in energy metabolism, while in the form of phospholipids it is a major structural component of cell membranes. In terms of systemic P concentrations, the kidney is a major organ controlling homeostasis. The renal excretion rate of P is regulated by its concentration in serum, with regulatory mechanisms independent of parathyroid hormones [40].

The results presented in this paper suggest that early P supplementation is indicated in severely burned patients who receive nutritional support. Moderate doses can effectively increase serum P concentrations.

### 4.4. Potassium

According to the results of the present study, no changes in serum K levels were observed relative to the reference value in any of the study groups, although individual patients from group 2 (moderate burns) had significantly elevated serum K concentrations. Similar findings were presented in a recent report of a study conducted among patients with electrical burns. Despite severe skin and muscle injury, they rarely showed hyperkalemia during the first 24 h after injury. Furthermore, hyperkalemia was shown to be independent of the severity of rhabdomyolysis or %TBSA [41]. High K levels are regarded as one of the pathologies associated with burns, but there is little research on this subject. In a study of 30 patients, elevated serum K and reduced serum Na levels were observed for the first three days after burn injury, but contrary to the literature data, they were not significant [42].

Potassium is a major cation involved in the conduction of nerve impulses and muscle contraction. As much as 98% of body K is found in intracellular fluid and only 2% in extracellular fluid. K homeostasis is regulated primarily by the kidneys, notably through aldosterone action. Derangements in K regulation and resultant changes in its serum concentration may alter membrane excitability and exert profound effects on the nerve, muscle, and cardiac function [43]. Hyperkalemia in burn patients is often explained to be a result of extensive superficial tissue injury, erythrocyte breakdown, and acidification of the body—with K released to increase pH [41].

In view of the results presented in this paper and the few previous reports, it can be concluded that the serum K level in burn patients is often an individual feature, not always correlated with the level of sustained injury. More cohort studies should be undertaken to improve our understanding of the mechanisms and causes underlying the presence or absence of K disorders in burn injuries. Given the important role of this cation in the human body, it is recommended that burn patients have their levels checked frequently.

### 4.5. Sodium

There were no statistically significant changes in serum Na levels relative to the reference value in any of the study groups or between study groups (Figure 1). Hypernatremia in burn patients is associated with intensive initial fluid resuscitation. It occurs within the first few days of injury and may be associated with an increased risk of mortality [44]. In addition, early hypernatremia may affect wound healing and skin graft take [45]. However, more recent data suggest that even subtle changes in plasma Na concentration can affect survival outcomes in critically ill patients [46,47]. Hypernatremia may have a greater impact on mortality than hyponatremia [47]. Several retrospective studies showed mortality rates of 30–48% in patients with severe hypernatremia >150 mmol/L [48]. In patients with critical burns, hypernatremia was a common condition, occurring in up to 11% of severely burned patients. In the end, it was concluded that dysnatremias may be an indicator of poor outcomes and increased mortality in severely burned patients, and the etiology of the abnormalities is multifactorial [49].

Sodium is the principal extracellular cation and the most osmotically active ion in the body. Therefore, Na metabolism is tightly regulated at the cellular and organ levels. Sudden alterations in plasma Na may be a consequence of various interventions, such as intravenous fluid administration or medication. They may indicate altered physiology and predict a rapid decline in health. In critically ill patients, dysnatremias are among the most common electrolyte abnormalities. They are also associated with increased mortality in critically ill patients [50]. The kidneys play a significant role in regulating Na concentrations in the body, and disorders in its metabolism result in abnormal sodium levels and consequent fluid imbalance between the tissues and plasma [51]. Therefore, incorporating Na measurement with other clinical indicators of patient status may improve the accuracy and precision of determining the clinical course of a severely burned patient.

### 4.6. Iron

Serum Fe levels in all the groups of burn patients in this study were significantly elevated compared to the reference value. While the highest level was noted in group 3, with severe burns, there were no statistically significant differences between individual groups. Such a rapid increase in serum Fe is consistent with the findings made by Sanchez-Agreda et al. [52]. In their study, the authors concluded that it was most likely related to severe hemolysis and release of Fe from erythrocytes. In further follow-up, after approx. 48 h, there was a sudden decline in Fe levels, which persisted for about a fortnight, despite frequent transfusions [52]. One likely explanation for this may be that Fe ions are used in reactions aimed at maintaining the oxidant/antioxidant balance. Severe burns cause a release of inflammatory mediators, including reactive oxygen species (ROS) and reactive nitrogen species (RNS), which eventually lead to local and remote pathophysiological effects. One of the key enzymes involved in the body’s antioxidant defence is catalase (CAT), known for its ability to convert harmful hydrogen peroxide into oxygen and water. Fe ions are well-known to be an integral component of CAT [53].

Bernát et al. [54] hypothesised about the possible iron sequestration in the reticuloendothelial system due to significant alterations in Fe kinetics in burn injuries. It was also suggested that impaired iron status may increase the risk for developing septic complications. There is a probable correlation between low serum levels of Fe and transferrin, with downregulated production of the latter in the liver, likely due to the enhanced amino acid catabolism characteristic of the hypermetabolic response. Under these conditions, Fe supplementation is unlikely to significantly stimulate erythropoiesis [54]. In addition, normal iron handling mechanisms are frequently altered in critically ill burn patients. The resulting hypoferremia may contribute to a generalised inflammatory state. Ceruloplasmin (Cp) is an acute-phase protein important in the regulation of Fe metabolism. It oxidises ferrous iron to the ferric form, which is less reactive and facilitates binding to ferritin, an iron storage protein. Iron deficiency in inflammation can be attributed, among others, to an early decrease in Cp oxidase activity. Patients with critical burn injuries are also at an increased risk for iron toxicity following blood transfusions, caused by a deficit of Fe-binding proteins and spontaneous ROS-producing reactions (Fenton reactions) catalysed by iron. Stress hypoferremia, i.e., a low blood iron status, is common in critically ill burn patients irrespective of the severity of injury, blood transfusion status, surgical procedures or sepsis [55].

Low serum Fe concentrations in burn patients have also been documented, leading to the development of anemia [56,57]. A low iron-binding capacity and low serum concentrations of transferrin may be implicated in the changes observed in the first week post-burn. A high level of soluble transferrin receptor (sTfR) may be a reliable indicator of iron deficiency anemia in hospitalised burn patients [58].

There is ample clinical evidence to suggest that impaired resistance to infection and inflammatory response may be directly proportional to higher stores of ferritin-bound iron within the body. This element status appears to play a significant role in the course of viral or bacterial infections. It is believed that hypoferremia confers relative resistance to various types of infections; and so iron deficiency might be a protective adaptive response [59]. There is a need for more research dedicated to iron levels in burn patients, their role at the individual stages of therapy, and the implications of high and low iron status, as the available findings are insufficient and sometimes inconsistent. In light of the above, caution is advised with respect to iron regulation in burn patients, especially at the early stages of treatment.

### 4.7. Zinc

The results of the present study show a slight decrease in serum Zn levels compared to the reference value in all the study groups, albeit with all results staying above the lower limit of normal. In their 1976 study, Lafargue et al. [35] reported that despite the increased excretion of zinc in the urine of burn patients, its serum levels remained within the reference range, a phenomenon which has not been fully explained to date. An altered zinc-protein binding activity in serum and Zn excretion in the form of a specific complex have been suggested as the potential causes [60]. In pediatric burn patients, Zn concentrations in wound exudates were observed to significantly exceed plasma concentrations, suggesting that the primary route of Zn loss might be through burn wound exudative losses. Standard hospitalisation often fails to restore normal Zn levels in plasma [61]. In addition, in the course of acute-phase reactions, Zn and other trace elements are rapidly redistributed from the bloodstream into bodily organs, e.g., the liver—as reported in an animal study [62], while serum levels of Zn-binding proteins drop [63]. Unfortunately, normal Zn concentrations in serum are not easily restored with supplementation, which is less efficient than in the case of Cu and Se supplementation. Nevertheless, with sustained adequate supplementation, the zinc status in burned tissues gradually improves, which is associated with improved wound healing [21].

Contrary to the above, no evident differences in serum zinc levels in burn patients receiving Zn supplementation vs. placebo were reported by Barbosa et al. [64]. While in theory, Zn supplementation in burn patients offers a number of benefits, improved wound healing is the only one to be well-documented in the literature. Cander et al. demonstrated the important role of serum Zn levels in critically ill patients, though not specifically burn-related [65]. The authors investigated the hypothesis that the decline in serum zinc concentrations among critically ill patients may be related to the length of stay in the ICU, organ failure and mortality. Their findings supported the conclusion that organ failure and critical illness contribute to a decline in serum Zn concentrations and that administration of zinc may help reduce mortality rates among critically ill patients [65]. In their 2019 paper, Olson et al. [66] reported on their pioneering study assessing the clinical impact of normalising serum Zn levels in patients with severe burns. They concluded that personalised supplementation of Zn failed to improve clinical outcomes during hospitalisation and hence fixed-dose supplementation without determining serum Zn levels may be preferrable [66].

It is worth noting that the acute-phase response may confound the interpretation of plasma Zn concentrations in burn patients. Both our results and the literature data indicate that the inflammatory state, with the onset of hypercatabolism, hypermetabolism and fluid shifts, is accompanied by a decrease in the concentrations of negative acute-phase proteins, such as albumin and prealbumin, while positive acute-phase proteins, like CRP, rapidly increase. Considering zinc binding to cellular proteins, its serum levels will continue to decline as long as the inflammatory state is present [67,68].

### 4.8. Copper

The results of our study show a slight decrease in copper concentrations in the serum of burn patients relative to the reference value in all study groups, albeit with all results staying above the lower limit of normal. These results appear to be consistent with previous reports on exudative losses of this mineral [21]. Major burns have been reported to be associated with low Cu levels, while serum copper determinations may fail to reflect the total losses of the mineral through wound exudation. Exudates account for approx. 20% of the mineral provided through supplementation [69]. Serum Cu levels were also observed to be inversely correlated with the burn surface area. Gosling et al. reported hypocupremia (<0.7 mg/L) in 48% of the studied patients in the first week after burn injury. Serum Cu concentrations in patients with burns accounting for less than 15% of the body remained normal throughout the study period [70].

The gentle decline in serum Cu concentrations in the early post-burn period may be attributed to the activation of the body’s compensatory mechanisms and the release of copper from tissue reserves. It is usually not until later on in treatment that symptoms of hypocupremia are observed [20], hence nutritional interventions should be implemented early on to minimize future deficits of Cu, with their consequent symptoms including impaired immunity, delayed wound healing and cardiac arrhythmia [71,72]. Given the fact that in Gosling’s study hypocupremia in one of the patients, whose burns covered 78% of the body’s surface, only resolved on day 75, after wounds had healed, it may be advisable to monitor Cu status in burn patients over the long term [70].

### 4.9. Selenium

Numerous studies show that burn patients experience a significant decline in serum Se levels, which has also been observed in this study and which may have a significant impact on treatment and recovery [73,74,75,76]. As a component of many enzymes, Se is involved in, among other things, the regulation of antioxidant enzymes (glutathione peroxidase, GPx), thyroid hormone metabolism, immune response [77] and wound healing [78]. In addition, Se is a structural part of a large group of selenoproteins that are essential for normal body function [53]. Severe selenium deficiency has been linked to cardiomyopathy, chronic osteoarthritis, immune function impairment and cognitive decline, as well as enhancing the risk of autoimmune thyroid disease. The consequences in the acute phase of severe burns, on the other hand, include exacerbation of oxidative stress, infectious complications, organ failure, and ultimately higher mortality [79]. Serum Se concentrations may be depleted due to higher losses of selenoprotein P (a major reservoir of selenium in plasma) through capillaries in the burn area [80].

The Se redistribution process is not well known. Intraperitoneal injection of endotoxin in rats caused a 30% decline in plasma Se after 16 h, with an increase of about 10% in the liver and skeletal muscles [81]. An interdependence was observed between reduced serum Se levels and increased CRP in various pathological conditions, including burns, compared to healthy individuals [82]. A similar relationship can be observed in the results presented here, although this correlation was not supported statistically. Selenium at physiological levels is involved in the inhibition of transcription factor NF-kappaB whose activation incudes inflammatory cytokine production. Se depletion, on the other hand, stimulates CRP production in hepatocytes during the acute-phase [82].

Literature data suggest that with sufficiently early supplementation of Se normal plasma levels can be easily restored, however frequent monitoring of plasma concentrations is recommended due to the high risk of overloading [21].

### 4.10. Chromium

Trivalent chromium (Cr III) is an essential nutrient for physiological glucose, protein and fat metabolism. It interacts with nucleic acids and proteins forming complexes participating in intracellular redox pathways [35]. A 20% loss of chromium through wound exudation (3.13 ± 1.1 mg/day) has been reported in clinical studies, but this was not accompanied by a decline in serum levels in the first week following a burn injury. In the present study, the Cr level did not decline over the first post-burn day, either.

Chromium deficiency has been suggested to lead to insulin resistance, a very common complication of severe burns, due to the role played by the mineral in glucose metabolism. According to current data, burn patients do not require additional Cr supplementation [80].

Glucose levels have been seen to markedly increase in patients with severe burns, which may be stimulated by increased glucagon levels. This is supported by the blood glucose determinations reported in this study, which are directly proportional to the surface area and severity of the burn (Table 2). The insulin-resistance inducing effect of acute pain has been confirmed in many studies and attributed primarily to its effects on glucose metabolism, with elevated levels of glucagon, growth hormone and cortisol frequently observed in burn patients [18,83]. Some papers have reported on the important role played by Cr in insulin function, improving blood glucose control and maintaining lean body mass [84].

The elevated Cr levels observed on the first day after burn injury in all the groups in this study may be due to the mobilisation of Cr from serum for redistribution to the injured tissues and in order to counteract the high blood glucose levels in the studied patients. A similar observation was reported in a rat study [85], where a rapid increase in muscle Cr concentration was observed on the first day after burn (after 6 h). It consistently declined over subsequent days. Early mobilisation and utilisation of Cr in muscle was accompanied by hyperinsulinemia, hyperglycemia, and a strong hormonal stress response. The increase in Cr concentration on the first day after burn injury may therefore be part of the stress response mechanism to burn trauma, as one way to prevent loss of lean body mass during the initial phase [86].

### 4.11. Manganese

Manganese is a co-factor for the antioxidant superoxide dismutase enzyme MnSOD (along with copper and zinc, playing a similar role in Cu/ZnSOD), which among others scavenges the superoxide anion into hydrogen peroxide and oxygen [53]. In this study, a marked (though statistically insignificant) increase in serum Mn levels was observed in all the study groups compared to the reference value. There are few studies dedicated to the investigation of serum Mn levels in critically ill patients and the effects of abnormal Mn status [86]. Friedman et al. studied a group of patients fed a purified diet deficient in Mn over an extended period and found its deficiency in serum [87]. They made no conclusions, however, as to whether low serum Mn reflects an actual whole-body deficiency. The few studies investigating serum Mn levels found the percentage of burn patients with low Mn to be marginal. Even with daily supplementation of trace elements, no changes were observed in the observation period [69,88].

The results presented in this study show a large increase in serum Mn levels across all the study groups. This is difficult to interpret given the observations made by Jafari et al. [69] and Lee et al. [88], who found no significant changes in Mn concentrations. However, it is important to mention that in our study we also observed a slight increase in serum ALAT and ASAT levels (Table 2), which may suggest some disturbance of liver metabolism in these patients and an increase in Mn levels, which under physiological conditions would be normalised by the hepatobiliary system [86].

According to the scarce available literature, there appears to be no need for routine Mn supplementation in patients with no deficiency. Still, patients undergoing long-term should be monitored regularly for Mn levels, notably in the case of severe burns.

### 4.12. Nickel

The results of the present study showed a marked (though statistically insignificant) increase in serum Ni concentrations in burn patients relative to the reference value. While insignificant, the observed trend seems interesting for a number of reasons. In the available literature on trace element concentrations in burn patients, Ni is neglected. There are virtually no reports on its levels and potential role in the therapeutic process. Neither is it accounted for in feeding regimens.

Ni is known to play an important role in erythropoiesis. It has been shown to stimulate erythropoietin production together with Co, by activating hypoxia-inducible transcription factor (HIF-1α). Nickel is also considered to be synergistic to Fe by promoting its intestinal absorption, while Ni deficiency can lead to iron deficiency and anemia [77]. Perhaps the high serum Ni concentration is somehow related to elevated Fe concentration observed across all the studied patient groups. In addition, some sources indicate that Ni can significantly affect the metabolism of other trace elements, such as Cu, Zn, and Mo. This has been demonstrated, e.g., in studies on goats and rats, where changes in the distribution of these elements were observed in the liver and kidneys of the test animals [89]. In another study, nickel was shown to bind best to albumin, followed by L-histidine and α-2-macroglobulin, which may explain to some extent the observed changes in serum Zn and Cu levels due to their enhanced excretion [90].

It is unclear why such a marked increase in serum Ni levels was observed in burn patients. Ni toxicity in humans is known to be affected by route of exposure, dose, and compound solubility. Ni usually enters the body by inhalation, but can also be ingested or absorbed by dermal contact. The main organs burdened by Ni toxicity are the kidneys and lungs, and to a lesser extent the liver, spleen, and heart [91]. It seems possible that burn patients have an increased exposure to Ni compounds, which may be present in large quantities in smoke (victims are very often in contact with it—inhalation exposure) and various types of burnt material (dermal route of exposure).

### 4.13. Aluminium

The present study demonstrated a marked increase in serum Al concentrations in burn patients relative to the reference value. The differences between the individual groups were not statistically significant. A study by Klein et al. was one of the few to describe Al accumulation in serum and bone in adults with burns exceeding >40% TBSA, finding Al deposition in the outer layer of bone as early as 8 days after the burn. It was suggested that minimizing Al loading in patients may accelerate their recovery from burn injury [92].

Severely burned patients experience changes in bone metabolism, involving a significant reduction in bone formation. The causes underlying these changes may be multifactorial, but Al loading, which also occurs in burn patients, has been noted to produce this type of injury in both humans and animals. Cutaneous exposure to Al appears to be the greatest concern, but the dynamics of Al entry into the bloodstream via a damaged skin barrier are still unclear, while enteral Al exposure is no greater than daily dietary exposure [93]. It is important to note that Al often contaminates various fluids used in intravenous therapy. Albumin, which is excreted primarily by the kidneys, can be highly contaminated with Al. Burn patients receive large amounts of albumin preparations, while often also suffering from renal impairment, which hypothetically exposes them to aluminium loading. In one study, an attempt was made to assess the risk of such loading. Aluminium levels were analysed in 12 burn patients. The serum levels were elevated in 8 of 12 participants, of whom 3 had toxic levels. However, no direct relationship was found between serum Al and the amount of albumin received. On the other hand, the patients with the highest serum Al levels had the most severe burns and none of them survived. Thus, burn patients receiving albumin may potentially be at risk for additional Al loading. Additionally, impaired renal function contributes to Al retention in the body [94]. The results of our study appear to confirm the few previous reports finding that Al can be a significant burden for burn patients. It is particularly noteworthy that high Al concentrations were observed as early as on the first day after burn injury, irrespective of the group to which the patients were classified. Given the small body of work dedicated to the aluminium status of burn patients, it would be advisable to undertake more extensive monitoring of this element at subsequent time intervals and look at the potential effects of Al in the therapeutic process.

## 5. Conclusions

Burn injuries, accompanied by a systemic inflammatory response, metabolic changes triggered by oxidative stress and often extensive would exudation, lead to a considerable loss of macro- and microelements, including essential trace elements, which in turn is associated with complications including e.g., greater susceptibility to infections and impaired wound healing [21,95]. This can be addressed with improved nutrition in terms of quality, quantity and diversity [96]. The studies to date have focused mainly on the imbalances in major trace elements, namely copper, selenium and zinc. Major depletion of these minerals in the early post-burn period is well acknowledged, and attributed to urinary excretion, fluid losses and, notably, burn wound exudation [72,97]. There is good reason to dedicate research to those three trace elements, given their special role in wound healing [98,99], antioxidant systems [100] and immune function [97]. Yet, a more comprehensive approach is needed for a proper investigation in a clinical setting of many other trace elements, likewise involved in immune function, regulation of gene expression, antioxidant defence, etc. [78]. Given the scarce and inconsistent literature data originating from relatively few, small and often dated studies, additional studies to understand the role of macro- and microelements in burn patients are needed for purposes including bringing up to date local nutritional protocols.

## Figures and Tables

**Table 1:** Classification of burn severity (according to [18]).

Group 1—Minor Burns (*n* = 14; W = 5, M = 9)	Group 2—Moderate Burns (*n* = 13; W = 2, M = 11)	Group 3—Severe Burns (*n* = 35; W = 6, M = 29)
≤15% TBSA in adults ≤10% TBSA in children and elderly patients ≤2% TBSA full-thickness burn in children or adults without functional or esthetic risk involving eyes, ears, hands, feet and perineum	15–25% TBSA in adults plus <10% full-thickness burns 10–20% TBSA partial-thickness burn in children under 10 years and adults over 40 years having <10% full-thickness burns ≤10% TBSA full-thickness burn in children or adults without functional or esthetic risk involving eyes, ears, hands, feet and perineum	≥25% TBSA ≥20% TBSA in children under 10 years and adults over 40 years ≥10% TBSA full-thickness burn all burns representing a risk of functional or esthetic impairment or disability of the eyes, ears, hands, feet and perineum all high-voltage electrical burns all burns complicated by major trauma or inhalation injury all burn patients with poor prognosis

TBSA—total body surface area.

**Table 2:** Selected blood parameters determined in patient groups 1–3 (G1, G2, G3).

	Ref. Range	Patients						Statistical Significance
		Group 1—Minor Burns		Group 2—Moderate Burns		Group 3—Severe Burns		
		Mean	±SD	Mean	±SD	Mean	±SD	
Total protein [g/dL]	6–8	6.654	1.382	6.457	0.824	5.953	1.010	G1 vs. G2—*p* = 0.044 G1 vs. G3—*p* = 0.0007 G2 vs. G3—*p* = 0.004
Albumin [g/dL]	3.5–5.5	4.304	0.471	4.189	0.498	3.428	0.798	G1 vs. G2—*p* = 0.044 G1 vs. G3—*p* = 0.0007 G2 vs. G3—*p* = 0.004
AST [U/L]	5–40	26.607	13.312	32.093	19.606	53.893	49.549	G1 vs. G3—*p* = 0.018
ALT [U/L]	5–40	21.361	12.753	26.804	26.223	33.607	19.021	G1 vs. G3—*p* = 0.013 G2 vs. G3—*p* = 0.039
Creatinine [mg/dL]	0.7–1.4	0.775	0.170	0.841	0.292	0.977	0.434	
Glucose [mg/dL]	70–115	118.167	27.663	122.411	28.686	165.650	97.119	
CRP [mg/L]	0.08–3.1	37.209	47.726	31.563	41.703	36.900	44.124	
IL-6 [pg/mL]	1.56–3.08	31.676	31.947	125.764	132.291	185.104	264.473	G1 vs. G2—*p* = 0.024 G1 vs. G3—*p* = 0.040
Hematocrit [%]	37–51	42.046	3.350	42.642	9.372	47.011	6.452	G1 vs. G3—*p* = 0.005
Hemoglobin [g/dL]	7.5–18	14.154	1.371	14.400	3.308	15.926	2.234	G1 vs. G3—*p* = 0.007

**Table 3:** Spearman rank correlations.

	Total Protein [g/dL]	Albumin [g/dL]	AST [U/L]	ALT [U/L]	Creatinine [mg/dL]	Glucose [mg/dL]	CRP [mg/L]	IL-6 [pg/mL]	Hematocrit [%]	Hemoglobin [g/dL]
**Ca**	NS	NS	0.27	NS	NS	NS	NS	NS	NS	−0.24
**Mg**	NS	NS	0.26	NS	NS	NS	NS	NS	NS	NS
**Mn**	NS	NS	NS	NS	NS	NS	NS	NS	NS	NS
**P**	0.26	NS	0.29	NS	−0.28	NS	NS	−0.33	NS	NS
**K**	NS	NS	NS	NS	NS	NS	NS	NS	NS	NS
**Zn**	NS	NS	NS	NS	NS	−0.33	NS	NS	NS	NS
**Cu**	NS	NS	NS	NS	NS	NS	NS	NS	NS	NS
**Fe**	NS	NS	NS	NS	NS	NS	NS	NS	NS	NS
**Na**	NS	NS	NS	NS	NS	NS	NS	NS	NS	NS
**Cr**	NS	NS	NS	NS	NS	NS	NS	NS	NS	NS
**Ni**	NS	NS	NS	NS	NS	NS	NS	NS	NS	NS
**Sr**	NS	NS	NS	NS	NS	NS	NS	NS	NS	NS
**Al**	NS	NS	NS	NS	NS	NS	NS	NS	NS	NS

NS–not significant.

## Data Availability

Restrictions apply to the availability of these data. Data was obtained from patient cards with the consent of the Hospital Authorities.
